# Investigating the correlation between genotype and phenotype in Prader-Willi syndrome: a study of 45 cases from Brazil

**DOI:** 10.1186/s13023-024-03157-2

**Published:** 2024-06-20

**Authors:** Hiago Azevedo Cintra, Danielle Nascimento Rocha, Ana Carolina Carioca da Costa, Latife Salomão Tyszler, Silvia Freitas, Leonardo Abreu de Araujo, Lisanne Incoutto Crozoe, Luísa Ribeiro de Paula, Patricia Santana Correia, Leonardo Henrique Ferreira Gomes, Letícia da Cunha Guida

**Affiliations:** 1 Laboratório de Alta Complexidade, Instituto Nacional da Saúde da Mulher, da Criança e do Adolescente Fernandes Figueira, Fiocruz, Rio de Janeiro, Brazil; 2 Unidade de Pesquisa Clínica, Instituto Nacional da Saúde da Mulher, da Criança e do Adolescente Fernandes Figueira, Fiocruz, Rio de Janeiro, Brazil; 3 Instituto Estadual de Diabetes e Endocrinologia Luiz Capriglione, Rio de Janeiro, RJ Brazil; 4 Centro de Genética Médica, Instituto Nacional da Saúde da Mulher, da Criança e do Adolescente Fernandes Figueira, Fiocruz, Rio de Janeiro, Brazil

**Keywords:** Prader-Willi syndrome, Imprinting, Epigenetics, Methylation-specific multiplex ligation-dependent probe amplification, Genotype, Clinical manifestations

## Abstract

**Background:**

Prader-Willi syndrome (PWS) is a genetic disorder characterized by abnormalities in the 15q11-q13 region. Understanding the correlation between genotype and phenotype in PWS is crucial for improved genetic counseling and prognosis. In this study, we aimed to investigate the correlation between genotype and phenotype in 45 PWS patients who previously underwent methylation-sensitive high-resolution melting (MS-HRM) for diagnosis.

**Results:**

We employed methylation-specific multiplex ligation-dependent probe amplification (MS-MLPA) and Sanger sequencing, along with collecting phenotypic data from the patients for comparison. Among the 45 patients, 29 (64%) exhibited a deletion of 15q11-q13, while the remaining 16 (36%) had uniparental disomy. No statistically significant differences were found in the main signs and symptoms of PWS. However, three clinical features showed significant differences between the groups. Deletion patients had a higher prevalence of myopia than those with uniparental disomy, as well as obstructive sleep apnea and an unusual skill with puzzles.

**Conclusions:**

The diagnostic tests (MS-HRM, MS-MLPA, and Sanger sequencing) yielded positive results, supporting their applicability in PWS diagnosis. The study’s findings indicate a general similarity in the genotype-phenotype correlation across genetic subtypes of PWS.

## Introduction

Described in 1956, Prader-Willi syndrome (PWS, OMIM #176,270) is an inherited disorder characterized by signs, symptoms, and genetic factors [1]. This genetic syndrome is characterized by a combination of signs, symptoms, and underlying genetic factors. It is mainly characterized by hypotonia, low stature, hyperphagia with subsequent obesity, neuropsychomotor development delay, endocrine deficiency, and behavioral and cognitive abnormalities [2, 3]. PWS affects all sexes and races, with an estimated incidence of 1:10,000 to 1:25,000 live births [4].

The cause of this neurodevelopmental disorder is the lack of expression of paternal copies of genes located in the 15q11-q13 region. PWS was the first human disease to be related to imprinting disorders [5]. This genetic disorder can be caused by three different molecular mechanisms: (a) de novo deletions of the paternal copy of the 15q11-q13 region (70%); (b) maternal uniparental disomy (UPD), where there is the inheritance of two maternal alleles of chromosome 15, in contrast to the normal one allele from each parent (25%); and (c) imprinting center defects (ICD), where we can find mutations or microdeletions in this small chromosomal region (< 5%) [6–9].

Although knowledge of the molecular etiology is not necessary to establish the diagnosis, genetic causes influence recurrence risk and counseling for PWS. The risk of recurrence is low, 1% for deletion and UPD but 50% for ICD with microdeletion [10].

**Fig. 1:**
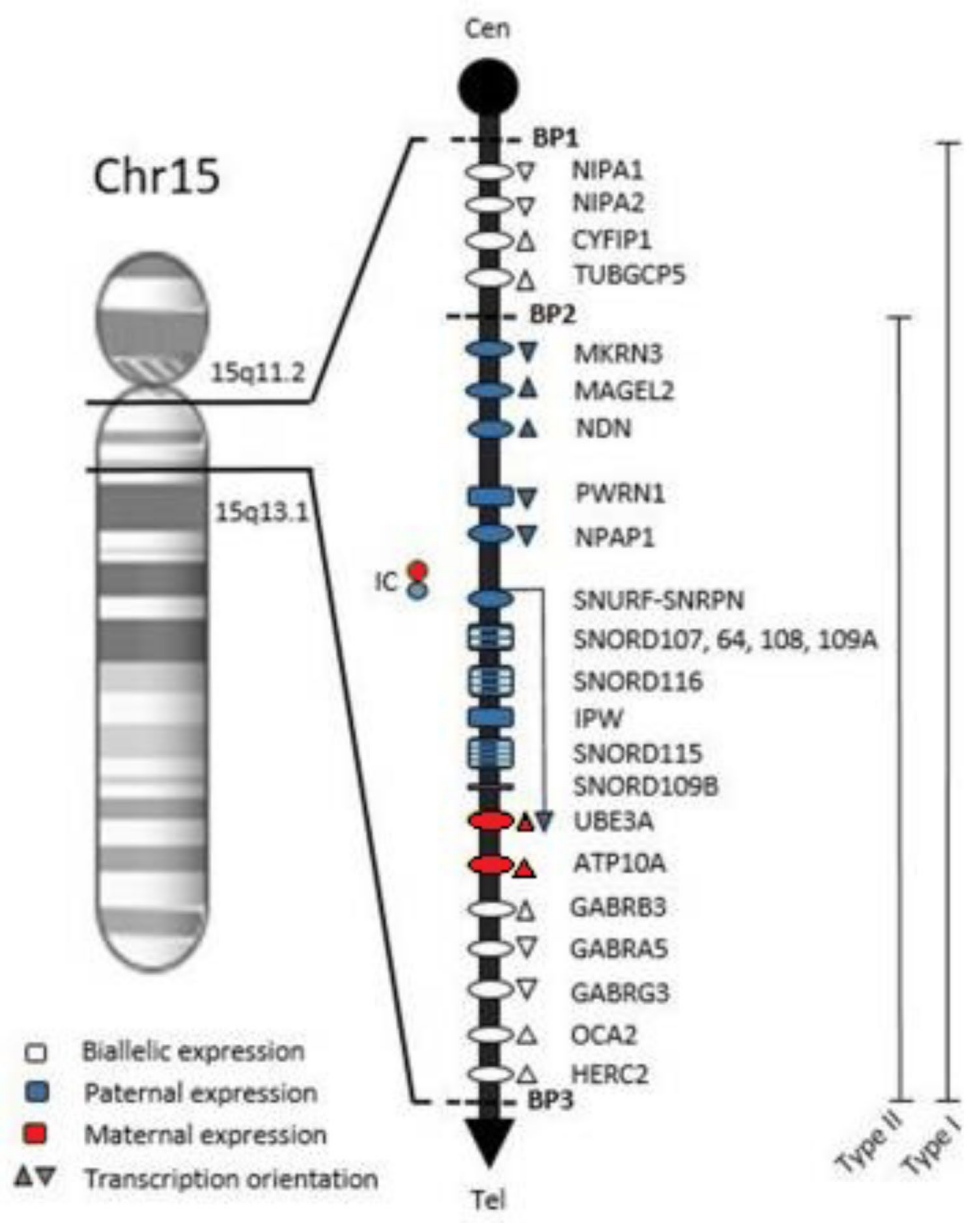
Chromosome map of the 15q11.2-q13.1 region. Symbols: ovals, protein-coding genes; rectangles, RNA genes; BP1, breakpoint 1; BP2, breakpoint 2; BP3, breakpoint 3; Type 1, BP1-BP3 deletion with ~ 6 Mb; Type 2, BP2-BP3 deletion with ~ 5.3 Mb; Cen, Centromere; Tel, Telomere; IC, Imprinting Center. Adapted from Costa et al. [13]

Comparisons of 15q11-q13 deletion versus UPD led to genotype-phenotype correlations. The PWS region is approximately 6 MB on chromosome 15 (Fig. 1). A type I (BP1/BP3) deletion is approximately 6 Mb, and a type II (BP2/BP3) deletion is approximately 5.3 Mb. Type I deletions cause severe problems, while type II deletions lead to less deviant behavior [11]. UPD is associated with a higher verbal intelligence quotient (IQ) and more psychosis and autism [12].

The clinical diagnosis of PWS is based on the clinical presentation and findings, which present difficulties during the neonatal and early childhood period. In this period, many of its signs and symptoms are nonspecific, and typical clinical features become apparent in later life stages. In parallel, the confirmation of the syndrome can only be given through laboratory diagnosis. There are many molecular strategies that evaluate the methylation status of the 15q11-q13 region, such as: Southern blotting, methylation-specific polymerase chain reaction (MS-PCR), postrestriction PCR of bisulfite-treated DNA, and methylation-specific multiplex ligation-dependent probe amplification (MS-MLPA) [14]. However, all these listed techniques are laborious, expensive and time consuming to obtain a diagnosis. Consequently, high rates of diagnostic errors and delays in interventions lead to a poor prognosis for these patients. It is essential to detect individuals with PWS in the neonatal period to initiate early intervention. Early treatment includes growth hormone (GH), which not only improves height and body composition (reduces body fat and increases muscle mass) but also decreases morbidity and mortality associated with obesity-related complications. Furthermore, it is of fundamental importance to study the correlation of the clinical phenotypes and their association with the genotype [15, 16]. Knowing the genetic mechanism that leads to the syndrome allows better preparation for the phenotypic complications associated with it.

The objective of this study was to increase the knowledge of genotype-phenotype correlations in PWS and thus contribute to the understanding of the wide clinical spectrum observed in PWS.

## Results

**Fig. 2:**
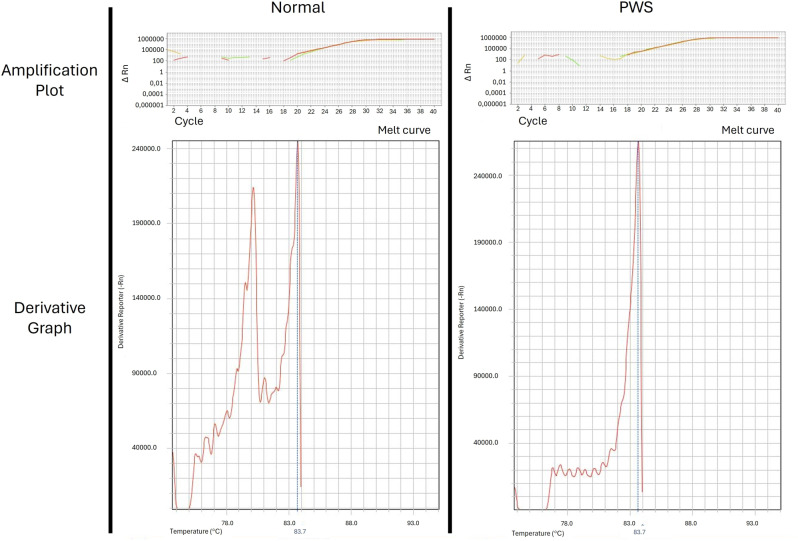
Allelic discrimination from the dissociation curve of amplified DNA from two individuals analyzed by MS-HRM. The left graph presents a normal control due to the presence of peaks related to the paternal and maternal alleles; the right graph shows PWS patient 01 with alteration in the methylation profile due to the presence exclusively of the maternal allele, characterizing PWS

Blood samples were collected from 166 individuals with clinical criteria for PWS and subsequently subjected to the Methylation-sensitive high-resolution melting (MS-HRM) protocol for molecular confirmation. Of these 166 individuals, 45 (40%) had confirmed PWS and were included in the study. The positive result for PWS in this protocol is due to a change in the methylation pattern, represented by the presence of a single peak relative to the maternal allelic dissociation temperature and the absence of the peak related to the paternal allele. The amplicons were differentiated by the temperature required for double-stranded DNA dissociation (78 °C for paternal and 83 °C for maternal alleles), identifying 121 (60%) of the 166 studied patients with a normal methylation profile due to the presence of two peaks related to maternal and paternal alleles (Fig. 2). The MS-HRM reactions of the 45 individuals showed little variation in the amplification cycle threshold (Ct), remaining constant at 25 (Fig. 2).

MS-MLPA was performed in 33 (73%). Of these, 18 (54%) were female and 15 (45%) were male. There were no discordant results between MS-HRM and MS-MLPA, with 45 patients diagnosed as having PWS and 121 non-PWS controls. Therefore, the concordance rate was 100%, and Cohen’s kappa was 1.0, indicating perfect agreement.

**Fig. 3:**
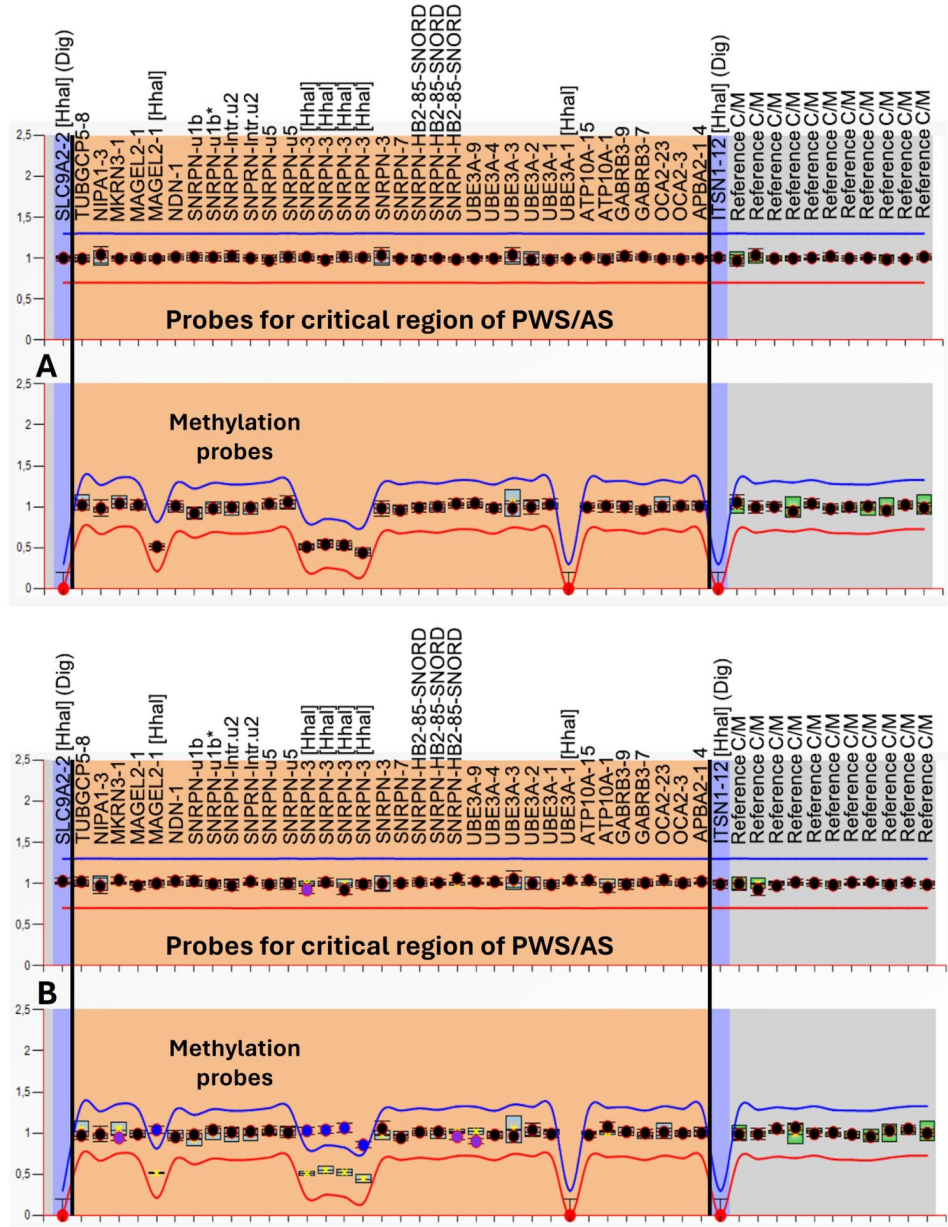
Graphic referring to the copy number analysis (upper panel) and methylation profile (lower panel) provided by Coffalyser.net software. The salmon-colored area comprises probes (represented by black dots) of interest for the 15q11-q13 region, including probes with sites for the Hha1 enzyme (represented by blue dots). (**A**) Normal control with a normal methylation status. (**B**) Nondeletion PWS.

**Fig. 4:**
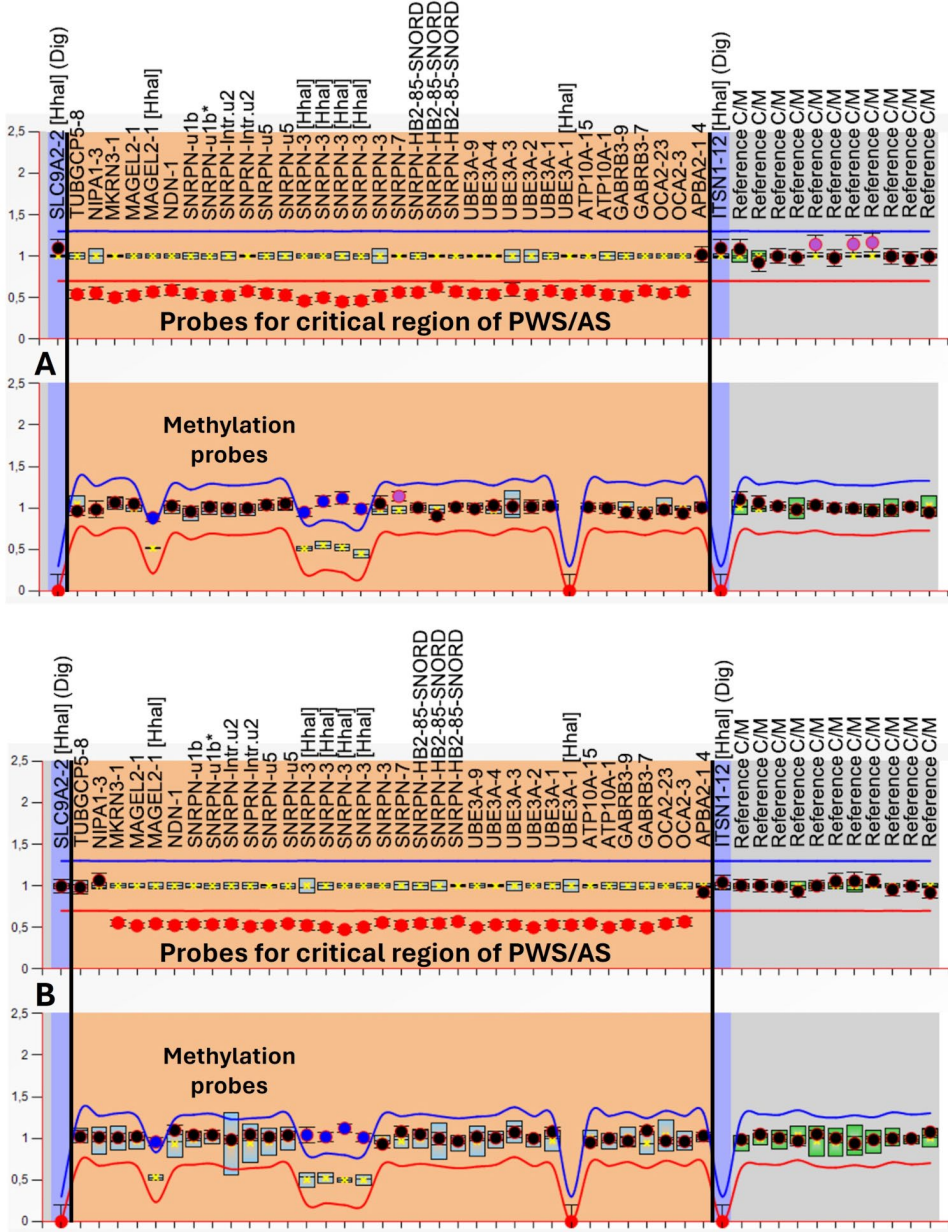
Graphic referring to the copy number analysis (upper panel) and methylation profile (lower panel) provided by Coffalyser.net software. The salmon-colored area comprises probes (represented by black dots) of interest for the 15q11-q13 region, including probes with sites for the Hha1 enzyme (represented by blue dots). (**A**) Type 1 deletion of PWS. (**B**) Type 2 deletion of PWS

Samples with a methylation status of approximately 50% (range between 40 and 60%) for specific PWS probes were identified as normal controls (Fig. 3A). In contrast, if this same status presented values close to 100% (greater than 80%), they were identified as the PWS pattern (Fig. 3B). In addition, the protocol allows differentiation of the deletion subtypes found in patients with PWS using the copy number data. The individuals with a deletion ranging from *NIPA1* to *OCA2* were identified as having type 1 deletions (Fig. 4A), while patients with a deletion ranging from *MKRN3* to *OCA2* were identified as having type 2 deletions (Fig. 4B).

Of the 33 individuals analyzed in our laboratory with MS-MLPA tests, 8 (24%) had type 1 deletions, 11 (33%) had type 2 deletions, and 14 (43%) did not have deletions, indicating UPD of chromosome 15 or defects in the imprinting center (Fig. 3B). No individual in the study showed any atypical deletions or Robertsonian translocations.

**Fig. 5:**
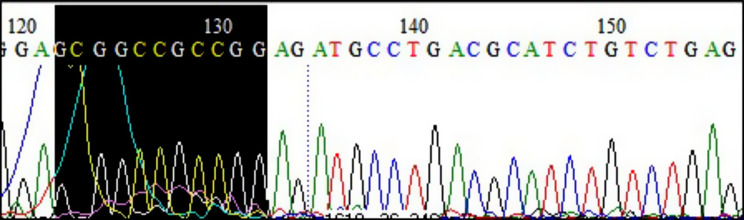
Representative electropherogram of the 15q11.2-q13 region derived from sequencing of PWS 13 patient. The black-marked sequence region indicates the CpG island found in the region

Out of a total of 45 patients, it was possible to identify the presence of deletions in 29 of them (64%) using the methodologies used in the study. The MS-MLPA method does not differentiate between cases of UPD and defects in the imprinting center. To investigate the etiology of these patients, Sanger sequencing of the promoter region of exon 1 of the *SNRPN-SNURF* gene was performed to evaluate base-pairing mutations and the epigenetic pattern of this region (Fig. 5).

**Fig. 6:**
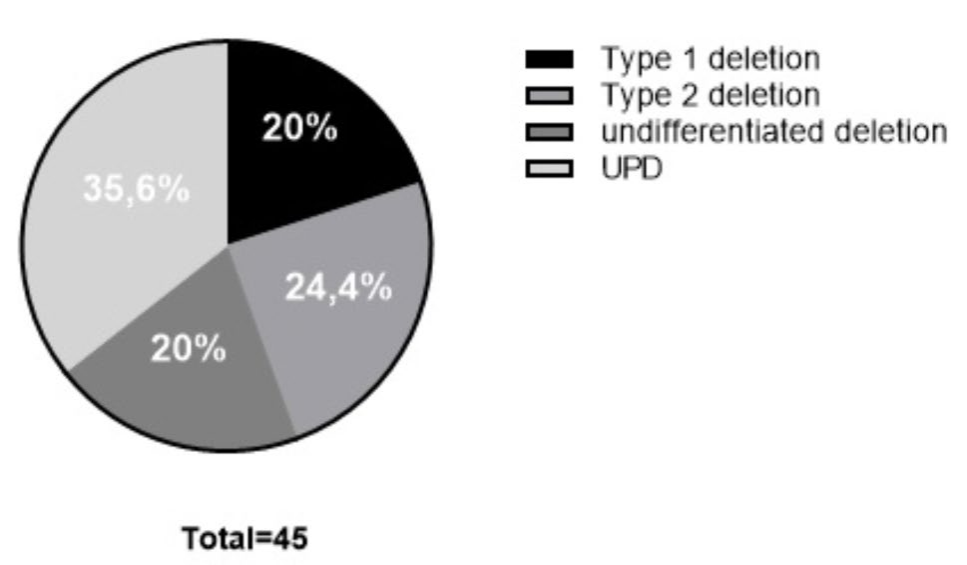
Distribution of molecular mechanisms in the 45 patients with PWS from this study

It was not possible to identify differences between the reference sequence deposited in GenBank (NG_012958.1), indicating UPD in all 14 individuals who did not show deletions in the MS-MLPA tests. Figure 6 summarizes the molecular mechanisms found in this cohort of patients.

We collaborated with the Luiz Capriglione State Institute of Diabetes and Endocrinology (IEDE/RJ) to obtain some of the clinical results of individuals in the study. IEDE was responsible for the clinical investigation.

The results on clinical history, physical findings, and neurological abnormalities are organized in Table 1. In our study, no significant differences were found between the two groups for the following data: reduced fetal activity, neonatal hypotonia, food-related behavioral problems, dental caries, scoliosis, type 2 diabetes mellitus, thick and viscous saliva, cryptorchidism, and hypoplastic labia minora.

**Table 1:** Clinical findings in patients with PWS divided by genetic subgroups

	Pacients with deletion (*n*=29)		Pacients with UPD (*n*=16)		P/a=0,05
	percentage	n	percentage	n	
Reduced fetal activity	82,8%	24	75%	12	P(2)=0,700
Polyhydramnios	27,6%	8	18,8%	3	P(2)=0,720
Breech position	37,9%	11	31,3%	5	P(1)=0,752
Short stature	55,2%	16	37,5%	6	P(1)=0,353
Failure to thrive in childhood	89,7%	26	68,8%	11	P(2)=0,111
Central obesity	51,7%	15	43,8%	7	P(1)=0,758
Dolichocephaly	34,5%	10	12,5%	2	P(2)=0,164
Narrow bitemporal diameter	58,6%	17	37,5%	6	P(1)=0,221
Almond-shaped eyes	86,2%	25	87,5%	14	P(2)=1,000
Upslanting palpebral fissures	34,5%	10	31,3%	5	P(1)=1,000
Thin upper lip	41,4%	12	18,8%	3	P(1)=0,189
Small-appearing mouth	48,3%	14	62,5%	10	P(1)=0,533
Downturned corners of mouth	44,8	13	62,5%	10	P(1)=0,353
Thick, viscous (reduced) saliva	31%	9	18,8%	3	P(2)=0,491
Enamel hypoplasia	10,3%	3	6,3%	1	P(2)=1,000
Early dental caries	31%	9	25%	4	P(2)=0,743
Dental crowding and malocclusion	20,7%	6	12,5%	2	P(2)=0,691
Strabismus	51,7%	15	25%	4	P(1)=0,118
Nystagmus	0%	0	6,3%	1	P(2)=0,356
Retinal hypopigmentation	0%	0	0%	0	-------------
Foveal hypoplasia	0%	0	0%	0	-------------
Hyperopia	3,4%	1	0%	0	P(2)=1,000
Myopia	31%	9	0%	0	**P(2)=0,017**
Hypernasal speech	41,4%	12	37,5%	6	P(1)=1,000
Weak or squeaky cry in infancy	62,1%	18	62,5%	10	P(1)=1,000
Hypoventilation	3,4%	1	0%	0	P(2)=1,000
Obstructive sleep apnea	51,7%	15	18,8%	3	**P(1)=0,055**
Feeding problems in infancy	51,7%	15	31,3%	5	P(1)=0,224
Gastroesophageal reflux	17,2%	5	31,3%	5	P(2)=0,455
Decreased vomiting	24,1%	7	31,3%	5	P(2)=0,728
Small penis	37,5%	6	16,7%	1	P(2)=0,616
Scrotal hypoplasia	56,3%	9	33,3%	2	P(2)=0,635
Cryptorchidism	81,3%	13	66,7%	4	P(2)=0,585
Hypoplastic labia minora	38,5%	5	20%	2	P(2)=0,405
Hypoplastic clitoris	23,1%	3	10%	1	P(2)=0,604
Scoliosis	27,6%	8	12,5%	2	P(2)=0,292
Kyphosis	3,4%	1	6,3%	1	P(2)=1,000
Small hands and feet	75,9%	22	75%	12	P(2)=1,000
Narrow hands with straight ulnar border	20,7%	6	12,5%	2	P(2)=0,691
Clinodactyly	24,1%	7	18,8%	3	P(2)=1,000
Hypopigmentation	20,7%	6	0%	0	P(2)=0,075
Blonde to light-brown hair	41,4%	12	18,8%	3	P(1)=0,189
Frontal hair upsweep	31%	9	12,5%	2	P(2)=0,279
Hyperinsulinemia	3,4%	1	18,8%	3	P(2)=0,121
GH deficiency	69%	20	50%	8	P(1)=0,336
Hypogonadotropic hypogonadism	10,3%	3	12,5%	2	P(2)=1,000
Diabetes mellitus (type 2)	3,4%	1	12,5%	2	P(2)=0,285
Skin picking	62,1%	18	50%	8	P(1)=0,534
Rectal picking	6,9%	2	18,8%	3	P(2)=0,330
Food related behavioral problems	58,6%	17	43,8%	7	P(1)=0,369
Temper tantrums	58,6%	17	56,3%	9	P(1)=1,000
Dificulty with transitions	24,1%	7	31,3%	5	P(2)=0,728
Stubbornness	55,2%	16	62,5%	10	P(1)=0,757
Obsessive behaviors	34,5%	10	37,5%	6	P(1)=1,000
Perseverant speech	48,3%	14	43,8%	7	P(1)=1,000
Obsessive-compulsive disorder	34,5\%	10	18,8%	3	P(2)=0,322
Psychosis	3,4%	1	0%	0	P(2)=1,000
Elopement	13,8%	4	12,5%	2	P(2)=1,000
Excessive daytime sleepiness	13,8%	4	37,5%	6	P(2)=0,131
Early-morning waking	55,2%	16	37,5%	6	P(1)=0,353
Night-awakening for food-seeking	6,9%	2	6,1%	1	P(2)=1,000
Several neonatal hypotonia	100%	29	100%	16	-------------
Poor neonatal suck and swallow reflexes	96,6%	28	93,8%	15	P(2)=1,000
Poor gross motor coordination	31%	9	25%	4	P(2)=0,743
Poor fine motor coordination	37,9%	11	31,3%	5	P(1)=0,752
Mild-to-moderate mental retardation	69,0%	20	43,8%	7	P(1)=0,122
Learning disabilities	51,7%	15	37,5%	6	P(1)=0,533
Increased risk of seizures	20,7%	6	18,8%	3	P(2)=1,000
Global developmental delay	82,8%	24	68,8%	11	P(2)=0,455
Speech-articulation problems	55,2%	16	62,5%	10	P(1)=0,757
Hyperphagia	51,7%	15	25%	4	P(1)=0,118
Temperature instability	27,6%	8	18,8%	3	P(2)=0,720
High pain threshold	48,3%	14	37,5%	6	P(1)=0,544
Unusual skill with jigsaw puzzles	48,3%	14	6,3%	1	**P(1)=0,007**

P(1)= Pearson’s chi-square test; P(2)= Fisher’s exact test; a=0.05. Lines without values are variables that had their analysis constant

Behavioral problems, such as stubbornness, excoriation disorder, temperamental issues, and food-related problems, were found in approximately 58% of patients with deletions and 52% of patients with UPD. No significant difference was found between the two groups regarding excessive daytime sleepiness and an increased risk of seizures.

Compared with normal individuals, patients with PWS have a delay in the development of motor coordination; however, in our study, over 30% of patients in both groups had deficits in gross and fine motor coordination. Global developmental delay was observed in 24/29 (82.8%) patients with deletions and 11/16 (68.8%) patients with UPD, but there was no significant difference between the groups.

Clinical phenotypes related to the ocular system were also evaluated. In 15/29 (51.7%) patients with deletion and 4/16 (25%) patients with UPD, they presented strabismus, but without a statistically significant difference between the two groups. Myopia was shown to be more frequent in patients with deletions than in patients with UPD (31% vs. 0%). In our study, only 1 patient in the deletion group had hyperopia, while only 1 patient in the UPD group had nystagmus.

Sleep integrity was evaluated through the manifestation of central sleep apnea, which was not observed in any of the patients. However, obstructive sleep apnea showed significant differences between groups, with this disorder present in 15/29 (51.7%) of patients with deletions and 3/16 (18.8%) of patients with UPD.

Regarding cognitive function, unusual puzzle-solving abilities that patients with PWS can present showed a significant difference between the groups, with a higher frequency in patients with deletions than in patients with UPD (48.3% vs. 6.3%).

As previously mentioned, PWS has some clinical signs and symptoms that are very characteristic of these patients. In our study population, severe neonatal hypotonia that improved with age was detected in all cases (45 individuals). Feeding problems in childhood, hyperphagia, hypogonadism, and mild dysmorphic signs, such as almond-shaped eyes, downturned corners of the mouth, thin upper lip, and small hands and feet, did not differ significantly in our study. The data are summarized below in Table 1.

## Discussion

Common molecular categories such as deletions and UPDs exhibit significant differences in clinical features. Paternal absence is associated with significant nutritional problems, sleep disturbances, depigmentation, speech impairment, and other symptoms [17]. Conversely, cases of UPD are often associated with prolonged gestation, increased verbal IQ, psychosis, and autism [18]. However, a UPD patient is less likely to have her PWS facies and pigmentation. To date, no genetic mutation has been described as a contributor to the devolopment of the syndrome. In a previous study, our group proposed an individualized analysis of genes and their role in the devolopment of clinical phenotypes. As has been demonstrated in this paper, the extensive clinical spectrum and the absence of a genotype phenotype specific correlation suggest that the multiple genes associated with PWS have an additive deleterious effect when deficiently expressed. Nevertheless, the lack of expression of the *SNORD116* gene cluster appear to be the best explanation for most of the PWS phenotype, although there is a need to investigate more of its mechanisms of action [13]. After that, the next step was to analyze if there was a genotype–phenotype correlation of our patients.

Clinical data from 45 PWS patients, mostly aged 1–28 years (mean 9 years), showed a frequency of 64.4% in deletion and 35.6% in UPD groups, slightly consistent with previous studies [7, 19]. Studies have also found an older maternal age within her UPD groups compared to deletions [4, 20, 21]. In particular, data on maternal age were not available in our study, however national statistics from DataSUS [22] show that between 2005 and 2020, maternal age increased annually in Brazil, especially in the 30–34 and 35–39 age groups, with increases of 28% and 62.6%, respectively. The higher frequency of UPD patients found in our study may be attributed, in part, to current trend of later pregnancies among women, which is a risk factor to the occurrence of UPD. In our cohort, we also conducted an analysis of the age at diagnostics, which ranged from 5 months to 23 years, with a median age of 1 year and 4 months, highlighting the crucial role of the reference laboratory in molecular diagnostics of PWS.

Regarding the possible influence of sex on patients with PWS, previous studies have shown discrepant results. Sanjeeva et al. [23] highlighted a higher prevalence of diagnoses in boys than in girls, while Gunay-Aygun et al. [24] reported a similar gender ratio. In our study, the gender ratio among PWS patients was approximately 1:1 (21 boys and 24 girls). Although our results do not manifest this difference between genders, certain specific signs, and symptoms of the syndrome, such as cryptorchidism, are more easily identifiable in boys. Establishing some level of complexity in the clinical diagnosis of these patients.

This study investigated the main clinical phenotypes in newborns with PWS, including severe neonatal hypotonia that improves with age (100%), almond-shaped eyes (86.6%), failure to thrive in childhood (82.2%), reduced fetal activity (80%), cryptorchidism (77.2%), and weak crying (62.2%). The results of WANG et al. [25] presented rates of hypotonia and weak crying higher than 95%, which is consistent with our data on hypotonia; however, the difference in results regarding weak crying may be explained by the fact that this clinical sign is subjectively judged by physicians.

Hypothalamic dysfunction underlies the major symptoms of patients with PWS [26, 27]. Symptoms of genital maldevelopment vary by gender, affecting the genitalia in men and the clitoris or genitalia in women. Male signs are evident at birth, whereas unlike male signs, female signs during the neonatal period can be obscured by obesity [28, 29]. In our study, 90.5% of 21 men had such symptoms, whereas only 29.1% of 24 women had such symptoms. Clinical observation bias could impact the analysis outcomes.

In this study, all cases of hypopigmentation were observed in patients with a deletion. However, this finding did not show a statistically significant difference in our analysis. We found a lower incidence of skin hypopigmentation (20.7%) in our study group compared to the 70–100% incidence of cutaneous hypopigmentation described in different studies [24, 30]. One possible explanation that should be investigated by us is the ethnic groups of the patients, as skin hypopigmentation is less easily identified among Caucasian individuals. The discrepancies can be attributed to (a) small sample sizes that are susceptible to selection bias or (b) the absence of a standardized clinical evaluation for skin hypopigmentation that is easily overlooked. Therefore, to determine if our results align with these studies, we need to increase the sample size, standardize the evaluation criteria, and conduct further research. The analysis of 54 cases of pediatric patients with PWS by Cassidy et al. [31] reported that the incidence rate of skin hypopigmentation in cases associated with deletion is much higher than that in UPD. Analyzing the structure of the genomic region of chromosome 15 associated with PWS, we observed the presence of the *OCA2* gene associated with type 2 oculocutaneous albinism, tyrosinase-positive. This gene is within the different breakpoints that lead to the appearance of two different deletion sizes.

In the present study, it was not possible to identify patients with defects in the imprinting center. The use of MS-MLPA confirmed the diagnosis by MS-HRM and in some cases allowed the identification of some deletions in 58% of the patients. Due to a limitation of the technique, individuals where deletions were not identified (52%) were analyzed indirectly for UPD and directly for the imprinting center defect.

In summary, hypotonia, feeding difficulties, and underdeveloped genital are general clinical features of PWS. Hypopigmentation of the skin, poor development of sex glands, specific facial characteristics, GH deficiency, and other characteristics described in Table 1 did not show significant differences between deletion and UPD.

Sinnema et al. [32] reported that patients with PWS have higher rates of maladaptive behaviors compared to other syndromes with intellectual disability. Webb et al. [33] suggested that UPD cases have milder maladaptive behaviors compared to deletion cases but have a higher risk of autism spectrum disorders and psychosis.

In our study, myopia showed a significant difference between the groups (*p* < 0.05). Myopia, following the diagnostic criteria established by Holm et al. [2], is considered a minor criterion and is associated with deletion. One possible molecular explanation is the loss of the *HERC2* gene. This gene belongs to the *HERC* gene family, which encodes a group of proteins that have various structural domains. All members have at least one copy of an N-terminal region showing homology to the cell cycle regulator RCC1, and a C-terminal HECT domain (homologous to the C-terminus of E6-AP) found in a series of ubiquitin protein ligase E3s. Genetic variations in this gene are associated with variability in skin, hair, and eye pigmentation. Several pseudogenes of this gene are located on chromosomes 15 and 16. This gene is also associated with the development of refractive abnormalities characterized by the ability to see near objects, which is associated with the phenotype of myopia [13].

Hypothalamic dysfunction is associated with clinical phenotypes such as hyperphagia and obesity in PWS due to hypometabolism. GH deficiency is common because GH affects IGF-I synthesis [34]. Regarding obstructive sleep apnea, a significant difference was observed, with a higher frequency in deletion cases than in UPD cases (*p* < 0.05). This condition may be associated with hypothalamic endocrine dysfunction that affects ventilatory control, leading to sleep-disordered breathing (SDB) [35]. From a molecular point of view, consultation with the Human Phenotype Ontology– HPO, using the key term obstructive sleep apnea, resulted in the association of genes and diseases present in the molecular region of PWS. For example, *MAGEL2* deletion has been associated with Prader-Willi-like syndrome (ORPHA ID:398,069), including a UPD case (ORPHA ID:98,754) with a role for the *SNRPN*, *OCA2*, *MAGEL2*, and *NDN* genes. Deletion outcomes vary by type (ORPHA ID: 98,793, ORPHA ID: 177,901), including type 2 genes such as *SNORD116-1*, *SNRPN*, *SNORD115-1*, *OCA2*, *MAGEL2*, *NDN*, and their role in the development of apnea. and the desired phenotype are revealed.

Despite research on PWS, little is known about its cognitive aspects. Patients with PWS often have mild to moderate intellectual disability, poor short-term memory, improved visual motor skills, and spatial awareness [18, 36]. People with PWS are known to have a special talent for putting puzzles together. Compared to other individuals with some form of intellectual disability reported, the parents of these patients had a greater pleasure their children had with puzzle assembly. Holm et al. [2], based on clinical impressions, included the unusual ability with puzzles as a supportive criterion for the clinical diagnosis of PWS. This unusual ability with puzzles can be explained due to the visual-motor strengths that many of these patients present, as well as their obsessive-compulsive tendencies, need for order, accuracy, and for things to be “just right” [37]. Dykens [18] was the first to examine clinical impressions that people with PWS have puzzle and word search abilities. In our study, this significant difference was observed more frequently in deletion cases than in DUP cases (*p* < 0.05). Consistent with our results, Dykens showed that DUM patients performed worse than paternal deletion patients with puzzle assembly abilities. Due to the lack of studies regarding this finding, little is still known about how the lack of information derived from the paternal allele on chromosome 15 is associated with puzzle assembly abilities or why they vary among genetic subtypes.

HOLM and colleagues [2] developed a preclinical form for identifying individuals with suspected PWS, considering age, and creating a scoring system for diagnosis. Observing the results of these studies, we see that individuals present major within the criteria and that differences in favor of deletion, such as myopia and obstructive sleep apnea, were observed in minor characteristics. Additionally, in our population, we see these differences in supportive criteria, where in favor of deletion, the unusual ability with puzzles was a statistically significant finding. This makes sense since the molecular screening (MS-HRM) and clinical diagnoses were consistent.

The silencing of the *MKRN3, NDN*, and *SNRPN-SNURF* genes present on maternal chromosome 15 is associated with specific CpG island methylation in the promoter region of these genes [38], while their copies on the paternal allele are unmethylated and expressed. Based on the different methylation statuses of *SNRPN-SNURF*, combining MS-HRM and MS-MLPA techniques yields good results in diagnosis and genetic counseling. With the increasing availability and use of molecular diagnostics in clinical applications, an increasing number of patients with PWS are diagnosed at an early stage. Currently, there are few studies of PWS in large samples of newborns with Brazilian genetic constitution. This pilot study included 45 cases of PWS in Brazilians and is able to delineate the major clinical phenotypes of PWS carriers in Brazil.

## Conclusion

The good performance of MS-HRM, MS-MLPA, and Sanger sequencing tests corroborates the applicability of a diagnostic strategy that can determine the genetic alterations present in individuals with PWS. Our findings in this study on genotype-phenotype correlation demonstrate that the genetic subtypes of the syndrome are generally similar, but some differences exist between subtypes.

According to our tests and statistical analyses, significant correlations were found between the genotype and phenotype of patients. Clinical signs such as myopia, obstructive sleep apnea, and unusual puzzle-solving ability were more frequent in patients with genetic deletions. Although the data are still limited for clear recommendations, it seems to be very promising, and more studies should be done to analyze this hypothesis.

## Methods

### Patients and samples

Blood samples were collected from 166 individuals with clinical criteria for PWS and subsequently subjected to the MS-HRM protocol for molecular confirmation. Of these 166 individuals, 45 had confirmed PWS and were included in the study. Patients were enrolled at the Fernandes Figueira National Institute of Women, Children and Adolescent Health (IFF/FIOCRUZ) and Luiz Capriglione State Institute of Diabetes and Endocrinology (IEDE/RJ). Blood samples from healthy individuals were collected as controls. This study was conducted in accordance with the Declaration of Helsinki and approved by the IRB of IFF (IRB approval number 45767015.0.0000.5269).

DNA extraction was performed using the PureLink Genomic DNA Mini Kit (Thermo Fisher Scientific, USA) following the manufacturer’s instructions. DNA quality was evaluated using a NanoDrop spectrophotometer, and its purity was also evaluated by the 260/280 and 260/230 wavelength ratios to avoid contaminants.

### DNA bisulfite treatment

Genomic DNA input was 250 ng/µl and was modified with sodium bisulfite using the EZ DNA Methylation-Lightning kit (ZymoResearch, USA). The modified DNA was quantified and evaluated with a NanoDrop spectrophotometer (Thermo Fisher Scientific, USA).

### Methylation-sensitive high-resolution melting (MS-HRM)

Using the bisulfite-treated DNA samples, we performed real-time PCRs as well as high-resolution melting (HRM) curve analysis using the Melt Doctor kit (Thermo Fisher Scientific, USA) and the 7500 Fast Real-Time PCR System (Thermo Fisher Scientific, USA) to confirm suspicions related to PWS. Each sample was analyzed in triplicate for MS-HRM. The primers used were described by Ribeiro et al. (2019, 2020) [39, 40].

PCR was performed in 200 µl PCR tubes with a final volume of 10 µl, containing 200 nmol/l of each primer, 5 µl of HRM-Master Mix (Thermo Fisher Scientific, USA), and 10 ng of bisulfite-treated DNA.

The initial denaturation (95 °C, 10 min) was followed by 40 cycles with a temperature of 95 °C for 30 s, followed by 60 °C for 60 s. After qPCR amplification, the PCR products were completely denatured at 95 °C, and the intensity of their fluorescence was monitored from 73 °C continuously to 85 °C with a thermal transition rate of 0.05 °C/s. Data analysis was performed in 7500 Fast Software v2.3 (Thermo Fisher Scientific, USA), with the derivative of fluorescence changes on the Y-axis and temperature (°C) on the X-axis.

### Methylation-specific multiplex ligation-dependent probe amplification (MS-MLPA)

Of the 45 patients, 33 were subjected to the MS-MLPA protocol using the SALSA MS-MLPA ME028 kit (MRC Holland, Netherlands). The 12 remaining patients were previously analyzed by MS-MLPA in another laboratory. The MS-MLPA experiment, and statistical analysis were performed according to the standard protocol previously described.

### Sanger sequencing

For DNA sequencing, PCR products were used as a template, with forward and reverse primers from the critical region of the PWS (Table 2) at a concentration of 3.2 pmol and the BigDye 3.1 sequencing kit (Applied Biosystems, USA). The reactions were analyzed on the ABI3730XL 96-capillary sequencer at the DNA Sequencing Platform (RPT01) of the PDTIS/FIOCRUZ program.

**Table 2:** Oligonucleotides used in Sanger sequencing

Primer	Forward (5’ > 3’)	Reverse (5’ > 3’)
*SeqPWS*	TACTTCCGGTGAGGGAGGG	AATAAAAGTGGCCGCTCCCC

### Statistical analysis of the data

Absolute and percentage frequencies of the phenotypic data seen in THE COMMITTEE ON GENETICS, 2011 [41] were compared by molecular subgroups of patients using 2 × 2 contingency tables. The relationship between molecular defects (paternal deletion or UPD) and phenotypic data was investigated using Pearson’s chi-square test (χ2) or Fisher’s exact test in cases where at least one expected frequency was less than 5. All statistical analyses were performed using SPSS software for Windows (Version 22), and the adopted significance level was 5%.

### Analysis of medical records

Medical records, including diagnosis, chief complaints, laboratory results, and other clinical information, were reviewed. For PWS, Holm’s diagnostic criteria were calculated [2].

## Data Availability

The datasets generated and/or analyzed during the current study are not publicly available due to the confidentiality and ethical aspects related to patient data but are available from the corresponding author on reasonable request.

## Abbreviations

IC: Imprinting Center
ICD: Imprinting Center Defects
MS-HRM: Methylation-sensitive High-Resolution Melting
MS-MLPA: Methylation-Specific Multiplex Ligation-dependent Probe Amplification
CT: Cycle Threshold
PWS: Prader-Willi syndrome
UPD: Uniparental Disomy
GH: Growth Hormone
PCR: Polymerase Chain Reaction
DNA: Deoxyribonucleic acid
IQ: intelligence quotient
IFF: Fernandes Figueira National Institute of Women, Children and Adolescents’ Health
IEDE: State Institute of Diabetes and Endocrinology Luiz Capriglione
DataSUS: Information Technology Department of the Brazilian Unified Health System
REM: rapid eye movement
HPO: Human Phenotype Ontology
µl: Microliter
Ng: Nanogram
°C: Degree Celsius
Pmol: Picomole
IPWSO: International Prader Willi Syndrome Organisation
